# Promoting Flight Crew Mental Health Requires International Guidance for Down-Route Quarantine Circumstances

**DOI:** 10.3389/fpubh.2022.854262

**Published:** 2022-03-04

**Authors:** Alpo Vuorio, Aedrian Bekker, Anna-Stina Suhonen-Malm, Robert Bor

**Affiliations:** ^1^Department of Forensic Medicine, University of Helsinki, Helsinki, Finland; ^2^Mehiläinen Airport Health Centre, Vantaa, Finland; ^3^Centre for Aviation Psychology, London, United Kingdom; ^4^Royal Free Hospital, London, United Kingdom

**Keywords:** aviation, pilot, mental health, quarantine, isolation, COVID-19

## Introduction

It is widely accepted that that during the COVID-19 pandemic, it is important to maintain international air routes not only for essential travel, but also for the transportation of cargo and medical and non-medical supplies. Modified regulations have been put in place for air crew during the pandemic for quarantine when traveling across borders. Quarantine is used for individuals who may have been exposed to the infective disease whereas isolation is of specific use in cases where an individual is infected with the disease. There is currently no international agreement or guidance, however, about how quarantine measures are carried out for airline crews. This pertains to, for example, accommodation conditions and standards, opportunities for communication and exercise facilities if quarantine is required locally during a lay-over. Anecdotal stories of air crew being housed in isolation in sub-optimum physical and mental health conditions, at some destinations, gives rise to concerns about their wellbeing and fitness to fly in some limited cases.

## Current Guidelines

Some general guidance has been issued by the International Civil Aviation Organization (ICAO) and International Air Transport Association (IATA) in relation to communicable diseases, including SARS-CoV-2 infection, in aviation (1, 2). ICAO recognizes that there may be different local practices regarding communicable disease management, but it is working toward harmonizing these practices especially during the COVID-19 pandemic (2). An additional example is published guidance on how to mitigate overseas layovers related to COVID-19 risk (3). According to this guidance, in countries with high risk of SARS-CoV-2 infection, landside overnight stays should be a maximum two nights and whilst self-isolation is recommended, food needs to be sourced within hotel.

## Need for International Guidelines

In this Commentary, we highlight the need for and importance of common international minimum standards for air crew required for quarantine to protect physical and mental health, and to ensure adequate rest in preparation for onward journeys. While flight crew may seem less vulnerable to mental health difficulties compared to the general population due to the exacting medical standards required to hold a Class 1 Medical Certificate, (4) it is possible that the local physical conditions and duration of COVID-19 related restrictions to include solitary quarantine, could impede mental health, (5) though aircrew vulnerability in quarantine has not been studied.

## Quarantine and Mental Health

In a recent systematic review of quarantine and mental health (6), 25 studies were analyzed. The results showed that mental health problems increased when quarantine or isolation lasted a week or longer and negative experiences were encountered [Odds ratios (ORs) for depressive disorders was OR 2.795 (95% CI 1.467–5.324), anxiety disorders OR 2.0 (95% CI 0.883–4.527), and stress-related disorders OR 2.742 (95% CI 1.496–5.027)]. Elevated levels of expressed anger among those forced to quarantine were also reported in this analysis. Additionally, quarantine has been associated with suicide (7), post-traumatic stress disorder (PTSD) (8) and deliberate self-harm (9).

In quarantine, the monotony of the environment can lead to hypo-stimulation and cumulative stress (7). Many factors affect the experience and coping with quarantine. The most important of these is the duration of quarantine (10) and the inadequacy of or apparent contradictions in the information provided by health authorities (11). Vital to meeting basic needs and ameliorating distress are access to a mobile telephone with connectivity. After the quarantine, management support has been found to be essential for successful return to work (8).

## Discussion

While airlines are responsible for mitigating the risk of exposure to COVID-19 infection during layovers, it is evident that there is need for quarantine in some cases. Based on medical literature the following factors presented below should be considered when designing international guidelines for minimizing the adverse effects of quarantine with flight crews (Table 1). Adequate provision of non-contradictory information is important, as is the physical environment in which those in quarantine are housed, fed and treated. Unimpeded remote access to families and loved ones is essential, as is support for those returning from trips involving quarantine.

**Table 1:** Proposed factors to minimize adverse effects of quarantine with flight crews.

**Factor**
Adequate information regarding rights and duration of quarantine by health authorities
Supplies (general and medical) provided
Explaining rationale for quarantine
Assistance those who are financially affected
Minimum requirements for physical conditions (fresh air, quality of food)
Possibility to be connected by telephone and internet
Possibility for daily physical exercise
Appropriate coping strategies delivered

*Based on (7, 12, 13)*.

Aeromedical examiners have a critical role in risk management when trying to minimize COVID-19 cases in aircrew (14). If COVID-19 cases occur among flight crew, occupational health care needs to provide support to aircrew in varying circumstances in a range of settings and locations. In the case of quarantine, airline occupational health care personnel need to actively assist aircrew on a daily basis.

While quarantine is essential in high-transmission areas following travel, it is recognized that those required to quarantine in the context of their work, such as air crew, are at higher risk of depression, anxiety, post-traumatic stress, deliberate self-harm, and suicide. The risk of deliberate self-harm and suicide may be elevated due to several stressors related to pandemic (15, 16). In the longer-term, PTSD is potentially related to increased risk of aircraft accidents (17). To reduce the harmful mental health risks associated with quarantine, it is recommended that basic internationally accepted minimum standards are applied to crew who must quarantine to promote mental wellbeing and prevent future mental health problems and an increased risk of unintended safety-related actions among crew.

## Author Contributions

AV and RB: writing the first draft. AV, RB, AB, and A-SS-M: editing to produce the final draft. All authors contributed to the article and approved the submitted version.

## Conflict of Interest

The authors declare that the research was conducted in the absence of any commercial or financial relationships that could be construed as a potential conflict of interest.

## Publisher's Note

All claims expressed in this article are solely those of the authors and do not necessarily represent those of their affiliated organizations, or those of the publisher, the editors and the reviewers. Any product that may be evaluated in this article, or claim that may be made by its manufacturer, is not guaranteed or endorsed by the publisher.
